# A Novel Missense Variant in Ultrarare SLC35A1-CDG Alters Cellular Glycosylation, Lipid, and Energy Metabolism Without Affecting CDG Serum Markers

**DOI:** 10.1155/humu/6290620

**Published:** 2025-06-26

**Authors:** Kristina Falkenstein, Lukas Hoeren, Frauke Kikul, Gernot Poschet, Christian Lüchtenborg, Ines B. Brecht, Ruth Falb, Darja Gauck, Tobias Haack, Andreas Hecker, Nastassja Himmelreich, Jürgen G. Okun, Britta Brügger, Christian Thiel

**Affiliations:** ^1^Pediatric I, Center for Pediatric and Adolescent Medicine, Medical Faculty of Heidelberg, Heidelberg, Germany; ^2^Biochemistry Center (BZH), Heidelberg University, Heidelberg, Germany; ^3^Plant Molecular Biology, Centre for Organismal Studies (COS), Heidelberg University, Heidelberg, Germany; ^4^General Pediatrics Hematology/Oncology, University Children's Hospital Tübingen, University of Tübingen, Tübingen, Germany; ^5^Institute of Medical Genetics and Applied Genomics, University of Tübingen, Tübingen, Germany; ^6^Center for Rare Diseases, University of Tübingen, Tübingen, Germany

**Keywords:** CDG-II, CMP-Neu5Ac transporter, congenital disorders of glycosylation, Golgi sialylation, protein instability, SLC35A1, SLC35A1-CDG

## Abstract

SLC35A1-CDG is a very rare type of congenital disorders of glycosylation (CDG) with only five cases known to date. Here, we review the literature and present new data from a sixth patient carrying the uncharacterized variant c.133A>G; p.Thr45Ala in the *SLC35A1* gene. In addition to known clinical symptoms of SLC35A1-CDG, the patient presents with failure to thrive, short stature, café-au-lait spot, and preauricular ear tag. Even though examination of CDG markers transferrin (Tf), alpha-1-antitrypsin (A1AT), and apolipoprotein CIII (ApoCIII) revealed no abnormalities in serum, the patient's fibroblasts showed significant alterations of protein expression or glycosylation of ICAM1, GP130, and TGN46 as well as differences in staining signals of lectins MAL-I, RCAI, and SNA and deviations in LC-MS analysis of total cellular N-glycans. Transfection of CRISPR/Cas9 generated *SLC35A1* HEK293 knockout cells with either wild-type *SLC35A1* or the c.133A>G variant restored the cellular CMP-Neu5Ac to wild-type levels, making a direct effect of p.Thr45Ala on the function of the transporter unlikely. Instead, our results imply that the residual transporter activity of 65% is caused by a decreased stability of the mutated SLC35A1 protein. Since O-GlcNAcylation was affected as well, energy and lipid homeostasis were analyzed and found to be significantly altered. Notably, proliferation and glycosylation of the SLC35A1-deficient patient fibroblasts were enhanced by supplementation of the cell culture medium with 10 mM GlcNAc.

**Summary**

• Normal routine congenital disorders of glycosylation (CDG) diagnostics do not rule out impaired protein function in patients with molecular genetic detection of *SLC35A1* variants.

## 1. Introduction

Sialylation, the process of attaching sialic acid residues to proteins and lipids, is fundamental to various biological functions. This modification influences molecular interactions, immune responses, and cellular signaling pathways. Altered sialylation patterns are associated with diseases such as cancer, immune disorders, neurodegeneration, and CDG. As a terminal modification on glycoconjugates, sialic acid affects molecular stability, receptor engagement, and host–pathogen interactions [1].

**Figure 1:**
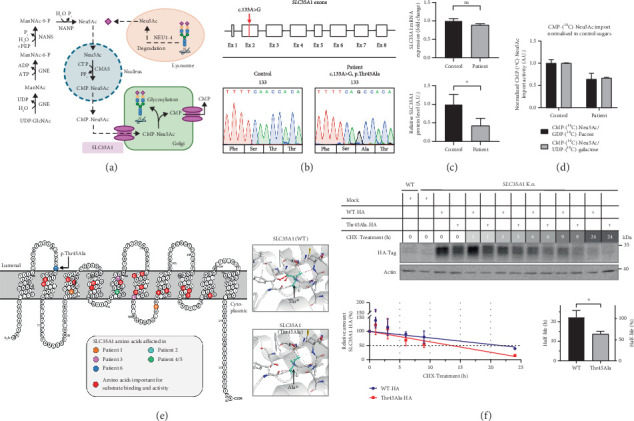
Effect of p.Thr45Ala on SLC35A1 expression and transporter activity. (a) Illustration of the cellular route of Neu5Ac. CMP-Neu5Ac is generated by the bifunctional UDP-*N*-acetylglucosamine 2-epimerase/*N*-acetylmannosamine kinase (GNE) converting UDP-GlcNAc to ManNAc and then to ManNAc-6-phosphate. Next, sialic acid synthase (NANS) and *N*-acetylneuraminate-9-phosphatase (NANP) convert ManNAc-6-phosphate to Neu5Ac via Neu5Ac-9-phosphate. The final step of CMP-Neu5Ac synthesis occurs in the nucleus, where Neu5Ac is linked to the nucleotide CMP by cytidine monophosphate *N*-acetylneuraminic acid synthetase (CMAS). CMP-Neu5Ac can also be generated through an alternative synthesis pathway, where sialic acid molecules that have been cleaved from glycoconjugates by the lysosomal neuraminidases NEU1-4 are recycled and subsequently reactivated with CMP in the nucleus. (b) Top: variant c.133A>G of Patient 6 localizes to Exon 2 of the SLC35A1 gene. Bottom: electropherogram (Sanger sequencing) of Exon 2 showing homozygosity for variant c.133A>G; p.Thr45Ala in fibroblasts of Patient 6. (c) Transcript and protein expression determined by qPCR and SLC35A1-ELISA revealed a normal mRNA level but significantly reduced SLC35A1 protein level in the patient's cells. (d) Measurement of the import activity of radioactively labelled sugar substrates into Golgi vesicles isolated from control and patient fibroblasts and normalisation of the CMP-[^14^C]-Neu5Ac import to the import of control sugars GDP-[^14^C]-fucose and UDP-[^14^C]-galactose. (e) Protein structure analysis. Left: visualization was performed by PROTTER (Version 1.0; http://wlab.ethz.ch/protter/start/). Highlighted in different colours are variants of the six known SLC35A1-CDG patients and of amino acid residues which are important for substrate binding and activity. Threonine 45 (blue circle) of Patient 6 is predicted to be located at the border of the first luminal loop to the second transmembrane domain. Right: due to the p.Thr45Ala variant (Patient 6), an interhelical H-bond is predicted to get lost. Simulation based on the murine Slc35a1 structure generated by DynaMut (PDB: 6XBO). (f) Cycloheximide (CHX) treatment of *SLC35A1* K.o. HEK293 cells expressing the wild-type (WT) or the mutated (p.Thr45Ala) SLC35A1-HA protein revealed a shorter half-life of the mutated SLC35A1. The relative amount of SLC35A1-HA was determined by quantification of the Western blot signals where the start of treatment was set to 100%. The regression lines were used to calculate the protein half-life (*y* = 50%).

For sialylation, CMP-Neu5Ac is required as a donor substrate. After its synthesis, it must be transported from the cytosol to the Golgi apparatus by the CMP-Neu5Ac/CMP antiporter SLC35A1. This transporter is a 37 kDa Golgi protein spanning 10 transmembrane domains (TMDs) (Figure 1e) and can form homodimers in vivo [2–4]. Additionally, it interacts with the Golgi *α*-2,3 sialyltransferase (ST3GAL4) [5], though other interaction partners may exist but remain unidentified.

The sialic acid residue derived from the donor CMP-Neu5Ac is typically terminally linked to an N- or O-glycan on a glycoprotein within the trans-Golgi by one of the 20 known sialyltransferases [6]. This process results in four possible linkages: Neu5Ac-*α*2,3-Gal, Neu5Ac-*α*2,6-Gal, Neu5Ac-*α*2,6-GalNAc, and Neu5Ac-*α*2,8-Neu5Ac [7].

Due to their terminal position and negative charge, sialic acid residues play a crucial role in glycoprotein stability by protecting them from proteolytic degradation. Furthermore, sialic acid residues are essential for numerous cellular interactions, including communication, regulation, adhesion, and immunological processes. Consequently, they are frequently found as posttranslational modifications on membrane-associated and secreted glycoproteins and glycolipids [8, 9]. Selectins, key cell adhesion molecules, recognize fucosylated and sialylated structures and are primarily expressed in endothelial cells, thrombocytes, and leukocytes. Leukocytes carry the tetrasaccharide Sialyl-Lewis X on their surface, which functions as a selectin ligand, facilitating leukocyte rolling [10]. However, terminal sialic acid residues on the cell surface can also serve as attachment sites for pathogens, such as the influenza virus [11] or bacteria like *Vibrio cholerae* [12] and *Pseudomonas aeruginosa* [13].

Given the extensive functions and biological significance of sialylation, it is not surprising that mutations in proteins involved in this modification typically result in multiorgan abnormalities in humans (GNE, OMIM #603824; NANS, OMIM #605202; and ST3GAL5, OMIM #604402). A nonfunctional SLC35A1 transporter causes SLC35A1-CDG (CDG-IIf, OMIM #605634), an ultrarare disorder with only five reported cases to date [14–17]. The pathological mechanisms resulting from CMP-Neu5Ac transporter deficiency are complex and not yet fully understood. Clinical phenotypes among affected individuals vary widely, ranging from mild neurological impairment and survival into adolescence to early childhood fatality. Common clinical manifestations include epilepsy, ataxia, microcephaly, neutropenia, macrothrombocytopenia, and recurrent infections.

Since the aberrant glycosylation patterns associated with this disorder do not always manifest in routine diagnostic tests, clinical diagnosis remains challenging, likely leading to undetected cases of SLC35A1-CDG. Here, we report the sixth patient with a novel missense variant in SLC35A1 and provide a comprehensive summary of all known cases, aiming to raise awareness of this rare glycosylation disorder.

## 2. Materials and Methods

### 2.1. Details of Ethics Approval and Patient Consent Statement

This work was performed in accordance with the Declaration of Helsinki and approved by the Ethics Committee of the University Hospital of Tübingen (Project No. 066/2021BO2). Written informed consent was obtained from the parents for genetic and biochemical analyses and for the use and publication of these data together with clinical information.

### 2.2. Cell Material

SLC35A1-CDG patient-derived fibroblasts, two commercially available (SSC, Millipore; HDF, Sigma-Aldrich), and one lab internal (N21) skin fibroblast control cell lines were used in this study.

### 2.3. Cell Culture

Fibroblasts and HEK293 cells were cultured in DMEM (Life Technologies) supplemented with 10% FCS (PAN-Biotech) and 1% Pen/Strep under 5% CO_2_ at 37°C. For preparation, cell monolayers with an 80% confluency were washed twice with ice-cold PBS and harvested by scraping in 5 mL PBS and centrifugation for 5 min at 2000 rpm and 4°C. Cell pellets were snap-frozen in liquid nitrogen and stored at −80°C until use.

### 2.4. WST-1 Cell Proliferation Assay

One day prior to measurement, 750 fibroblasts were seeded per well (96-well plate). The total volume was 100 *μ*L. Incubation was carried out at 37°C up to 10 days as indicated. Cell proliferation was addressed in a colorimetric assay by measurement of the conversion of WST-1 to formazan at 450 nm. Processing was carried out according to the manufacturer's instructions (CELLPRO-RO, Roche). For the GlcNAc supplementation assay, the same conditions as described above were used but with 10 mM GlcNAc as an additive to the cell culture medium.

### 2.5. Isoelectric Focusing (IEF) of CDG Marker Proteins

IEF was performed from patients' and controls' sera on a Phast System (GE HealthCare) as described [18].

### 2.6. LC-MS Analysis of Whole Serum and Fibroblast N-Glycans

LC-MS analysis of *N*-glycans from whole serum glycoproteins of a control pool (*n* = 120) and the SLC35A1-CDG patient was conducted by the GlycoWorks RapiFluor-MS *N*-Glycan Kit (Waters) on an Integrated UPLC-FLR/QTOF mass spectrometry system (BioAccord, Waters) as described [19]. Glycoprotein isolation from fibroblasts was performed according to the protocol of Hennig et al. [20] but with a starting concentration of 300 *μ*g total protein. The labelling of *N*-glycans after *PNG*ase F treatment was performed by using the GlycoWorks RapiFluor-MS *N*-Glycan Kit and was measured by usage of the BioAccord system. Data processing and normalization of migration times to a dextran standard were performed with UniFi software (Waters). *N*-Glycans were annotated based on migration time matching with the internal *N*-glycan database (Waters). Intensities of *N*-glycan peaks were calculated by normalizing to the sum of all assigned peaks. Detected fluorescence-labeled peaks were verified for accuracy by mass spectrometry. No quantitative intersample comparison was performed in this case. The detected individual glycans were grouped automatically using the internal UniFi software according to their classification as nonsialylated glycans, monosialylated glycans, disialylated glycans, trisialylated glycans, defucosylated glycans, antennary 1 glycans, antennary 2 glycans, antennary 3 glycans, antennary 4 glycans, and high mannose glycans.

### 2.7. Import of Nucleotide Sugars Into Golgi-Enriched Membrane Fractions

Import of nucleotide sugars (CMP-[^14^C]-Neu5Ac, GDP-[^14^C]-fucose, and UDP-[^14^C]-galactose) into Golgi-enriched membranes was determined as described previously [21].

### 2.8. Quantitative Real-Time PCR (qRT-PCR)

Total RNA was extracted from 1 × 10^6^ cells using the RNeasy Mini Plus Kit (Qiagen). The reverse transcription was performed with 1 *μ*g total RNA using the RevertAid First Strand Kit (Thermo Fisher) according to the manufacturer's protocol for random hexamer primers. qRT-PCR was performed as described previously [19]. Primers used for amplification of *SLC35A1* were h*SLC35A1*_qPCR_F (caaccacagccgtgtgtatca) together with h*SLC35A1*_qPCR_R (tgctaagagctaggaaagccat) and h*β-ACTIN*_qPCR_F (agagctacgagctgcctgac) together with h*β-ACTIN*_qPCR_R (agcactgtgttggcgtacag) for the reference gene *ACTINB*. Amplification levels were quantified regarding *β-ACTIN* and calculated relatively to the results from control fibroblasts.

### 2.9. SLC35A1-ELISA

The SLC35A1 protein level was determined by the usage of the human CMP-sialic acid transporter (SLC35A1) ELISA kit (Abbexa). For sample preparation, 3 × 10^6^ cells were washed three times with DPBS, harvested, and pelleted by centrifugation (5 min at 2000 rpm and 4°C). The pellet was resuspended in 250 *μ*L DPBS and sonicated (3 × 10 pulses). After centrifugation (13,000 rpm, 10 min, 4°C), the samples' total protein amount of the supernatant was adjusted with DPBS and subsequently diluted in a 1:10 ratio. Further steps of the ELISA kit were conducted according to the manufacturer's protocol. Each replicate was carried out in technical duplicates.

### 2.10. SDS-PAGE and Western Blot

SDS-PAGE and Western blotting were carried out by standard procedures with 10 *μ*g protein derived from patient and control samples. Primary antibodies for detection of SLC35A1 (Proteintech, BIOZOL, MyBioSource), ADAMTS13, ATP5H, GP130 (all Thermo Fisher), GAPDH, histone 2B (H2B), H2B-GlcNAc (all Abcam), ICAM1 (Sigma), OGA, OGT, TGN46 (all Proteintech), O-GlcNAcylation (Santa Cruz), and HA-Tag (Sigma) were used in 1:1000 dilution, *β*-ACTIN (Sigma) in 1:10,000 dilution, and incubated overnight at 4°C under constant movement. Secondary antibodies used were anti-rabbit IgG-conjugated with horseradish peroxidase (HRP) (for SLC35A1, ADAMTS13, H2B, H2B-GlcNAc, ICAM1, GP130, OGA, OGT, TGN46, and HA-Tag) from Dianova or anti-mouse IgG-HRP (for *β*-actin, GAPDH, O-GlcNAcylation, and ATP5H) from Santa Cruz in a dilution of 1:10,000. After adding Pierce ECL Western blotting substrate, the protein signal was detected by light emission on a Fusion SL4 detector (PEQLAB). ImageJ was used for quantification (http://imagej.nih.gov/ij/).

### 2.11. Lectin Blot

SDS-PAGE and blotting were carried out by standard procedures with 10 *μ*g protein derived from patient and control samples. As lectins, we used biotin-coupled *Ricinus Communis Agglutinin I* (RCAI), SNA, *Maackia amurensis* lectin (MAL-I), and succinylated (succ.) wheat germ agglutinin (WGA) (all from Vector Laboratories) in a dilution of 1:400. For detection, HRP-coupled streptavidin (Vector Laboratories, United States) diluted in 1:10,000 in TBST 0.1% was used.

### 2.12. Transfection of HEK293 Cells

HEK293 cells (1 × 10^6^) were seeded 1 day prior to transfection. FuGENE HD Transfection Reagent (Promega) was used according to the manufacturer's guidelines with 2 *μ*g of the respective plasmid DNA. Forty-eight hours after transfection, cells were scraped and lysed (if transiently transfected) or washed with PBS and further cultured in DMEM containing 250 *μ*g/mL neomycin (for stable transfection).

### 2.13. Generation of a HEK293 SLC35A1 Knockout (K.o.) Cell Line

Generation of a *SLC35A1* K.o. cell line was achieved by a CRISPR/Cas9 genome editing approach in HEK293 cells. As guide RNA (gRNA), a Sequence Targeting Exon 2 of the *SLC35A1* gene (5⁣′-CACCGGGTATAGACTGCAGCCATCA-3⁣′, described previously in Banning et al. [22]) was used (SeqLab-Microsynth GmbH, Göttingen, Germany) and cloned into the pSpCas9(BB)-2A-Puro plasmid according to the protocol provided by DKFZ Heidelberg (https://crisprflydesign.org/protocols/, high-level ubiquitous expression of a single Cas9 sgRNA).

HEK293 cells were transfected with pSpCas9(BB)-2A-Puro using FuGENE 4K Transfection Reagent (Cat #E5911, Promega, Fitchburg, Wisconsin, United States). Transfected cells were isolated to generate single-cell colonies and afterwards expanded and harvested for screening, using the Phire Animal Tissue Direct PCR Kit (Thermo Fisher, Waltham, United States). Gene editing of the *SLC35A1* gene was confirmed by the Sanger sequencing of the gRNA binding site using forward (5⁣′‐ACTAAGTAATGTCTTTGTTGCACG‐3⁣′) and reverse (5⁣′‐TGTTTAGCAGCATCCTTGGTC‐3⁣′) primers. The efficacy of the K.o. was verified by qPCR and SLC35A1-ELISA.

### 2.14. Cycloheximide (CHX) Treatment in Cell Culture

HEK293 cells were cultivated under standard conditions. CHX treatment was performed with 300 *μ*g CHX per milliliter medium over 1, 3, 6, 9, and 24 h chase, according to Kao et al. [23]. Further analysis of SLC35A1-HA expression was performed as described under Western blotting.

### 2.15. Amino Acid and Acylcarnitine Analysis

Amino acid and acylcarnitine composition in patient and control fibroblast homogenates (starting amount 100 *μ*g) were determined as described previously [24].

### 2.16. ATP and CMP-Neu5Ac Level Analysis

5 × 10^6^ cells were used for the analysis and processed according to the method of Røst et al. [25]. An ACQUITY I-Class PLUS UPLC system (Waters) coupled to a QTRAP 6500+ (AB SCIEX) mass spectrometer with electrospray ionization (ESI) source was used for the separation and detection of ATP and CMP-Neu5Ac. Data collection was performed using Analyst 1.7.2 (AB SCIEX) and processed using the OS Software Suite 2.0.0 (AB SCIEX).

### 2.17. Lipid Oil Analysis

The detection of neutral lipids was assessed in 5 × 10^4^ patient and control fibroblasts as described previously [26].

### 2.18. Lipid Analysis

Quantitative mass spectrometry lipid analysis was performed with 100 *μ*g from control and patient fibroblasts according to Özbalci et al. [27].

### 2.19. Statistics

All experiments were performed at least in triplicate except the LC-MS analysis of N-glycans isolated from whole serum and fibroblast lysate (*n* = 1) and the transporter activity assay (*n* = 2 for CMP-[^14^C]-Neu5Ac and GDP-[^14^C]-fucose import and *n* = 1 for UDP-[^14^C]-galactose import). Data were analyzed by GraphPad Prism using the Student's *t*-test for single comparisons or one-way ANOVA followed by Bonferroni's test for multiple comparisons. Patient's results of subsequent analyses were displayed as combined data and were compared to combined control data. Significances used were as follows: ∗*p* ≤ 0.05, ∗∗*p* ≤ 0.01, ∗∗∗*p* ≤ 0.001, and ∗∗∗∗*p* ≤ 0.0001. Data are displayed as means ± SD.

### 2.20. Trio Exome Sequencing

Trio exome sequencing and bioinformatic analyses were essentially performed as described previously [28]. In brief, exonic and adjacent intronic regions were enriched from the patient's and parental genomic DNA using a SureSelectXT Human All Exon V7 kit (Agilent Technologies, Santa Clara, California). Sequencing as 2 × 101 bp paired-end reads was performed on a NovaSeq 6000 (Illumina, San Diego, California), and generated sequences were analyzed using the megSAP pipeline (https://github.com/imgag/megSAP).

### 2.21. In Silico Prediction

For in silico prediction SIFT, SIFT4G and CADD were used. The structural prediction was performed using DynaMut (http://biosig.unimelb.edu.au/dynamut) [29].

### 2.22. Variant Interpretation

The pathogenicity of this missense variant was classified using the American College of Medical Genetics (ACMG)/Association for Clinical Genomic Science (ACGS) recommendations [30, 31]. The ClinVar accession number is SCV005326549.1.

## 3. Results

### 3.1. Clinical Description

The patient is an 8-year-old girl born at term to healthy, unrelated parents from Italy and Germany. A younger sister and brother are healthy. The patient's birth measurements and postnatal adaption were normal. At the age of 13 months, she presented with failure to thrive (length 70 cm [< 3rd centile], bodyweight 7.140 kg [< 3rd centile], head circumference 46.5 cm (15th centile), and *aphthous* mouth ulcerations). She furthermore had a history of recurrent bacterial upper respiratory tract and middle ear infections requiring antibiotic treatment. A febrile generalized tonic–clonic seizure lasting several minutes occurred once. Laboratory testing was remarkable for neutropenia (260/*μ*L, normal range (NR) 1800–6800/*μ*L) possibly in the context of autoimmune antibodies (weak positive for CD16b) and lymphocytosis (86%, NR 20%–45%) with fluorescence-activated cell sorting (FACS) showing a decreased natural killer cell count (CD16 + CD56+: 0.04 × 10^9^/L, NR 0.1–1.4 × 10^9^). In addition, mild anemia (10.7 g/dL, NR 10.8–12.8 g/dL) was documented, but there was no evidence of macrothrombocytopenia. Thrombocytopenia was only seen in early childhood. Physical examination revealed two pigmentary abnormalities resembling a palm-sized café-au-lait spot on the trunk and a smaller one in the axilla, an ear tag, and a small umbilical hernia but no further dysmorphic features. Although the girl showed signs of premature fatigue when walking at the age of 4, she had no motor impairments at the age of 8 years. In the further course, lactose intolerance was diagnosed, and cardiac examination revealed a hemodynamically insignificant patent foramen ovale (PFO). Regular subsequent checkups showed normalization of her blood counts and normal psychomotor development.

### 3.2. Clinical Synopsis of SLC35A1-CDG

An overview of the clinical characteristics of the known six SLC35A1-CDG patients is shown in Table 1. Seizures were reported in five of six patients of which two were tonic–clonic, one was versive, and two were not further specified. Four of six patients showed ataxia, delayed development (psychomotor in three and one was not further specified), and macrothrombocytopenia. Three individuals showed microcephaly, and hypotonia was named in two of six patients, presenting as generalized muscular (1×) and in the lower extremities (1×). Further symptoms were physical abnormalities, neutropenia, hemorrhagic difficulties, recurrent infections, and behavioral problems in two of six patients, respectively. Two patients deceased at ages 3 and 22, respectively. Routine CDG diagnostics showed abnormal results in four of six cases [14, 16, 17]. Notably, one of the deceased patients presented with a normal IEF pattern in diagnostics [15, 32].

### 3.3. Genetic Analyses

A search for rare (MAF < 0.1% in 1000 genomes, ExAC or gnomAD, in-house database) nonsynonymous DNA variants in genes that have been associated with the patient's clinical features prioritized a homozygous missense variant in Exon 2 of the *SLC35A1* gene (NM_006416.5; ENST00000369552.9:c.133A>G, p.Thr45Ala, Figure 1b) with the parents being heterozygous carriers. This allele has a frequency of 0.002775 in the gnomAD total population corresponding to 785 heterozygous and 3 homozygous carriers. While the presence of three homozygotes in controls usually questions a causal association of a given variant with severe pediatric phenotypes, the rather mild clinical presentation and benign course of the disease led us to pursue further experiments to investigate the functional relevance of the c.133A>G variant.

### 3.4. Variant Interpretation

The patient's change alters an evolutionarily highly conserved amino acid residue p.Thr45Ala and is accordingly predicted pathogenic in silico (SIFT: disease-causing [0.00] and CADD score [25.50]) (PP3). Our in vitro functional analyses (see below) validated the deleterious impact of the p.Thr45Ala variant on protein function (PS3). Additionally, the presence of SLC35A1-CDG was confirmed by the identification of hyposialylated N-glycans through LC-MS analysis in both fibroblasts and serum/plasma, via Western blot of specific glycoproteins, and was strengthened by the detection of abnormal lectin staining. Both threonine 45 (T45) and its surrounding amino acids (Y42, F43, S44, T46, and A47) are highly conserved across species (from humans to bearded dragons, Figure S1A) indicating a significant role of this peptide sequence for SLC35A1 function. Prediction tools (DynaMut, Phyre2, PROTTER) suggest that the polar T45 is positioned on the side of the Golgi lumen where it forms the last amino acid of the first luminal loop and is located at the transition to the hydrophobic region of the second TMD (Figure 1e, left). Since an interhelical hydrogen bond is lost due to the p.Thr45Ala variant, an impact on the molecule's flexibility is likely (Figure 1e, right). While the presence of homozygous carriers in controls usually questions a causal association of a given variant with severe pediatric phenotypes, the rather mild clinical presentation and benign course of the disease do not rule out a causal association with the observed biochemical phenotypes. The identified variant c.133A>G; p.Thr45Ala was accordingly classified as “likely pathogenic” (ACMG guidelines: PM2 [moderate], PP3 [supporting], and PS3 [strong]). We pursued additional biochemical experiments to further investigate the functional relevance of the c.133A>G; p.Thr45Ala missense variant.

### 3.5. Normal Transcript but Reduced Protein Level due to SLC35A1 Instability

qRT-PCR studies revealed a mild decrease in the *SLC35A1* transcript level in the patient cells compared to the controls only (control: 1.0 ± 0.06, patient: 0.89 ± 0.04; *p* > 0.05) (Figure 1, top). However, the protein level, determined by a SLC35A1-ELISA, showed that the signal intensity exhibited a significant reduction in comparison to the control samples (control: 1.0 ± 0.27, patient: 0.42 ± 0.19; *p* = 0.039) (Figure 1, bottom). To investigate whether the p.Thr45Ala variant affected the SLC35A1 stability, a CRISPR/Cas9 HEK293 *SLC35A1* K.o. cell line was generated and transiently transfected with the tagged constructs SLC35A1-WT-HA (K.o. + WT) or SLC35A1-A133G-HA (K.o. + A133G), respectively. Subsequently, the transfected cells, expressing the SLC35A1-WT-HA or SLC35A1-Thr45Ala-HA protein, were treated with the translation inhibitor CHX for a period of 0–24 h and harvested at various times, followed by detecting and quantifying the HA-tag signal. The wild-type protein showed a half-life of 22.2 ± 3.5 h (100% ± 15.8%), whereas the half-life of the mutated SLC35A1 was significantly decreased to 13.7 ± 1.4 h (61.6% ± 6.1%; *p* = 0.017) (Figure 1).

Furthermore, the transfected HEK293 *SLC35A1* K.o. cells K.o. + WT and K.o. + A133G revealed no striking differences in GP130 and TGN46 reglycosylation (Figure S1B), indicating a general functionality of the protein variant.

### 3.6. p.Thr45Ala Leads to Reduced CMP-Neu5Ac Transport Into the Golgi

Next, the import of CMP-[^14^C]-Neu5Ac into Golgi-enriched vesicles was determined. Normalized to the import of GDP-[^14^C]-fucose (control: 1.0 ± 0.08) and UDP-[^14^C]-galactose (control: 1.0 ± 0.01), the CMP-[^14^C]-Neu5Ac import in the patient was reduced to 0.64 ± 0.13 and 0.67 ± 0.01, respectively (Figure 1d), resulting in an average residual SLC35A1 (p.Thr45Ala) activity of 65.5%.

### 3.7. Elevated CMP-Neu5Ac Level in Patient Fibroblasts and HEK293 *SLC35A1* K.o. Cells

To elucidate whether the reduced transporter activity led to an accumulation of CMP-Neu5Ac, the CMP-Neu5Ac level was measured by UPLC-coupled ESI-MS/MS. Increased values were found in the patient's fibroblasts (patient: 1.91 ± 0.97, control: 1.0 ± 0.24; *p* > 0.05; Figure S1C, left), and significantly elevated amounts were measured in the HEK293 *SLC35A1* K.o. cells (K.o. Mock: 2.86 ± 0.65, WT Mock: 1.0 ± 0.22; *p* < 0.0001; Figure S1C, right). Notably, the values in the HEK293 *SLC35A1* K.o. cells returned to WT levels after transfection with the wild-type *SLC35A1* construct (K.o. + WT: 1.07 ± 0.12), suggesting a direct effect of SLC35A1 deficiency on the cytosolic CMP-Neu5Ac pool. Transfection with the plasmid carrying the mutated *SLC35A1* (K.o. + A133G: 1.27 ± 0.21) led to a similar result, further suggesting that the SLC35A1 (p.Thr45Ala) protein is still widely functional.

### 3.8. Differential Effects on Glycosylation in Serum and Fibroblasts

#### 3.8.1. Serum

IEF of CDG marker proteins (transferrin, alpha-1-antitrypsin, and apolipoprotein CIII) showed normal glycosylation patterns (data not shown). However, binding studies revealed signal reductions for *Sambucus nigra* lectin (SNA, binds to *α*-2,6 sialic acid residues) in the patient's serum to 71% ± 7% (*p* = 0.053), for MAL-I (binds to *α*-2,3 sialic acid residues) to 64% ± 9% (*p* = 0.039), and for WGA (binds to GlcNAc and *α*-2,3 sialic acid residues) to 57% ± 6% (*p* = 0.009) (Figure S2A). Remarkably, Western blot analysis of the highly glycosylated “A Disintegrin and Metalloprotease with Thrombospondin Type 1 Repeats, Member 13” (ADAMTS13) displayed a significantly reduced protein level (patient: 0.55 ± 0.15, control: 1.0 ± 0.14; *p* = 0.018) (Figure S2B). Furthermore, LC-MS analysis of N-glycans derived from whole serum glycoproteins identified 32 distinct sugar structures. In the patient's sample, a 34% increase in nonsialylated structures was observed, without a preference for a specific type of sialic acid linkage. The most pronounced effect was seen in trisialylated N-glycans, which were markedly underrepresented (−97%). Elevated levels were detected for sugar structures such as F(6)A2 (+50%), F(6)A2(6)G(4)1 (+32%), and F(6)A2G(4)2 (+23%). A major hallmark was the overall reduction of sugar moieties bound to glycoproteins, as exemplified by the 50% decrease in the disialylated biantennary complex-type N-glycan A2G(4)2S(6,6)2 compared to the control pool (Figure S2C,D and Table S1).

#### 3.8.2. Fibroblasts

**Figure 2:**
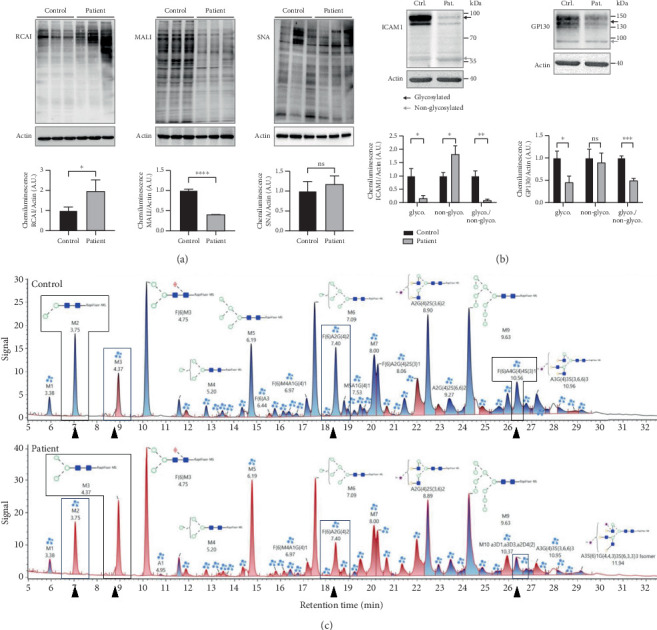
Effect of SLC35A1 deficiency on glycosylation in fibroblasts. (a) Lectin binding studies showed a significant increase of nonsialylated structures (RCAI) and a significant decrease in *α*-2,3 sialylated glycans (MAL-I) in the patient, whereas the amount of *α*-2,6 sialylated residues (SNA) in SLC35A1-CDG was comparable to controls. (b) Expression of ICAM1 and GP130 is significantly reduced in the patient's cells (Pat.) compared to controls (Ctrl.). For quantification, the fully glycosylated protein forms (glyco., black arrows) were compared to the hypoglycosylated forms (nonglyco, grey arrows). (c) LC-MS analysis of N-glycans in fibroblasts revealed a deviating amount of individual N-glycans in the patient's cells (indicated by black boxes and arrows).

Lectin staining by RCAI (binds to nonsialylated residues) further showed a significantly increased signal in the patient cell lysate (patient: 1.96 ± 0.57, control: 1.0 ± 0.17; *p* = 0.049; Figure 2a), indicating a reduced sialylation. SNA signal was unremarkable (patient: 1.17 ± 0.21, control: 1.0 ± 0.24; *p* > 0.05), whereas signal for MAL-I was significantly reduced in the SLC35A1 (p.Thr45Ala) patient (patient: 0.40 ± 0.002, control: 1.0 ± 0.03; *p* < 0.0001) (Figure 2a). Western blot analysis of glycosylation markers ICAM1 and GP130 (both Figure 2b) and TGN46 (Figure S3A) resulted in clearly abnormal signals in the SLC35A1-deficient fibroblasts. Calculated ratios of the glycosylated to the hypoglycosylated protein forms revealed significantly reduced values in case of the patient (ICAM1: 0.08 ± 0.05, *p* = 0.004; GP130: 0.49 ± 0.05, *p* < 0.001; and TGN46: 0.20 ± 0.01, *p* = 0.001) in comparison to the control (ICAM1: 1.0 ± 0.19, GP130: 1.0 ± 0.05, and TGN46: 1.0 ± 0.14).

In LC-MS analysis (Figure 2c and Table S1), 49 N-glycans were annotated. Interestingly, the proportion of nonsialylated N-glycans in the control cells (69%) was comparable to the patient cells (67%). However, shifts regarding the sialylated sugar structures were observed. The amounts of mono- and trisialylated N-glycans were found to be reduced in the patient cells (10% and 4%, respectively) in comparison to the control (14% and 7%, respectively), whereas the amount of disialylated glycans was increased in the patient (patient: 18%, control: 11%). Furthermore, and in contrast to the LC-MS N-glycan analysis performed in serum, alterations in the distribution of several species like Man2GlcNAc2 (M2; patient: 3%, control: 5%), Man3GlcNAc2 (M3; patient: 5%, control: 3%), F(6)A2G(4)2 (patient: 3%, control: 5%), and F(6)A4G(4)4S(3)1 (patient: 2%, control: 4%) (Figure 2c) were found. In addition, on mass spec level, the sialylated glycans A2G1S1 (m/z 1041.412 Da) had an 8-fold increase and A1G1S1 (m/z 857.076 Da) a 7-fold increase in the patient's fibroblasts. The results of glycan profiling thus reflect the limiting effect of SLC35A1 transport capacity on the sialylation process.

### 3.9. Reduced O-GlcNAcylation in SLC35A1-Deficient Fibroblasts

Since the glycosylation marker protein TGN46, which carries different N- and O-Glycans but also O-GlcNAc residues in its fully glycosylated form (predicted by DTU Health Tech, https://services.healthtech.dtu.dk/), showed a reduced height in the patient's cell lysate (Figure S3A), we wanted to elucidate whether O-GlcNAcylation in the patient's cells was affected by determining the expression of OGT and OGA. OGT was reduced in the patient compared to control fibroblasts (patient: 0.56 ± 0.26, control: 1.0 ± 0.13; *p* > 0.05), whereas OGA was slightly increased (patient: 1.22 ± 0.15, control: 1.0 ± 0.14; *p* > 0.05; Figure S3B, top left).

In addition, the expression of H2B and its Ser112 O-GlcNAcylated form (H2B O-GlcNAc) was quantified. In the lysate of the patient cells, the amount of H2B was comparable to the expression in the control samples (control: 1.0 ± 0.19, patient: 0.93 ± 0.05; *p* > 0.05); instead, the O-GlcNAcylated form of H2B was significantly reduced in the patient cells (control: 1.0 ± 0.06, patient: 0.68 ± 0.07; *p* = 0.023; Figure S3B, top right). The overall level of O-GlcNAcylated proteins was also significantly diminished in the patient cells, as demonstrated by signals of the O-GlcNAc antibody (control: 1.0 ± 0.18, patient: 0.38 ± 0.16; *p* = 0.043) and lectin staining with succ. WGA (control: 1.0 ± 0.21, patient: 0.65 ± 0.13; *p* = 0.043) (both Figure S3B, middle).

### 3.10. Metabolic Abnormalities in SLC35A1-Deficient Fibroblasts

**Figure 3:**
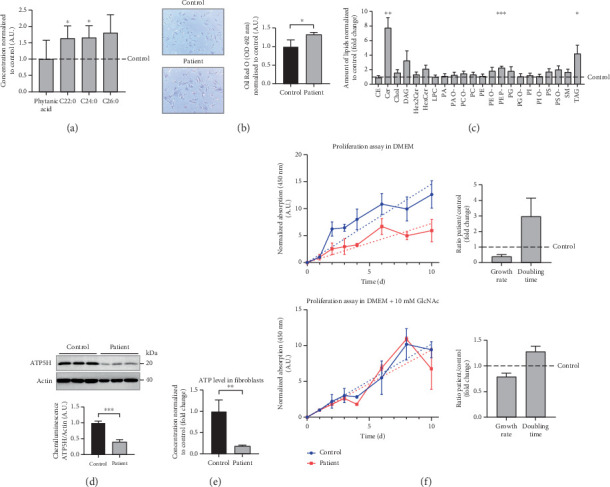
Metabolic measurements and GlcNAc supplementation assay in fibroblasts. (a) Measurement of very long-chain fatty acids (VLCFAs) in the lysate of patient fibroblasts normalized to the mean values of the control cell lysate revealed partially increased levels. (b) Staining of lipids in control and patient fibroblasts with Oil Red O presented with an increase in pronounced coloration in the case of the patient's cells indicating an accumulation of neutral lipids. (c) Nanoelectrospray ionization tandem mass spectrometry of cellular lipids revealed significant alterations concerning ceramide (Cer), phosphatidylethanolamine plasmalogen (PE-P), and triacylglycerol (TAG) within the main lipid classes. Abbreviations: CE, cholesteryl ester; Chol, cholesterol; DAG, diacylglycerol; PC, phosphatidylcholine; PE, phosphatidylethanolamine; PI, phosphatidylinositol; PS, phosphatidylserine; SM, sphingomyelin; Hex2Cer, dihexosylceramide; HexCer, hexosylceramide; LPC, lysophosphatidylcholine; PA, phosphatidic acid; PG, phosphatidylglycerol. (d) Reduced expression of ATP5H combined with a decreased ATP level (e) hint to a general mitochondrial impairment in SLC35A1-CDG. (f) Reduced proliferation due to decreased growth rate and increased doubling time in the patient's cells can be improved by 10 mM GlcNAc as a cell culture supplement.

Analysis of amino acids and acylcarnitines (C0-C18OH) revealed normal values (data not shown). However, the determination of concentrations of very long-chain fatty acids (VLCFAs; C20–C26:0) showed an increase in C22:0–C26:0 in the patient cells, with C22:0 (control: 1.0 ± 0.28, patient: 1.64 ± 0.38; *p* = 0.036) and C24:0 (control: 1.0 ± 0.27, patient: 1.66 ± 0.37; *p* = 0.028) being significantly elevated (Figure 3), indicating a disturbance of peroxisomal *β*-oxidation. Lipid Oil Red O staining further indicated a significantly higher amount of neutral lipids in the patient cells (1.33 ± 0.05) compared to control fibroblasts (1.0 ± 0.18; *p* = 0.039) (Figure 3). Subsequent analysis of lipid classes by nano-ESI tandem mass spectrometry revealed widely normal levels for most lipid classes. Notably, the amount of the glycerolipids diacylglycerol (DAG) and triacylglycerol (TAG) was increased in the patient cells, with TAG being significantly elevated (patient: 4.20 ± 1.17, control: 1.0 ± 0.49; *p* = 0.045), in accordance with the result of the lipid Oil Red O staining (see 6.12.3). In addition, the levels of ceramide (Cer; patient: 7.74 ± 1.41, control: 1.0 ± 0.08; *p* = 0.002) and phosphatidylethanolamine plasmalogen (PE-P; patient: 2.26 ± 0.23, control: 1.0 ± 0.08; *p* = 0.001) were significantly increased in the patient fibroblasts (Figure 3) (Table S2). Furthermore, the expression of the mitochondrial marker protein “H^+^ transporting, mitochondrial Fo complex subunit D” (ATP5H), a subunit of the ATP synthase, was significantly reduced in SLC35A1 (p.Thr45Ala) cells (patient: 0.40 ± 0.07, control: 1.0 ± 0.06; *p* < 0.001) (Figure 3). This reduction may be associated with the significantly decreased ATP level observed in the patient cells (patient: 0.18 ± 0.02, control: 1.0 ± 0.27; *p* = 0.004) (Figure 3).

### 3.11. Enhanced Proliferation of SLC35A1-Deficient Fibroblasts With GlcNAc Supplementation

Under standard cell culture conditions, the patient cells exhibited a significantly reduced growth rate compared to control fibroblasts (ratio patient/control: 0.39 ± 0.13-fold) leading to a significantly increased doubling time (ratio patient/control: 2.97 ± 1.18-fold) (Figure 3f). The addition of 10 mM GlcNAc to the culture medium resulted in an improved proliferation of the patient's cells with the ratio of patient to control growth rate increasing to 0.79 ± 0.07-fold and the doubling time decreasing to 1.28 ± 0.11-fold, which nearly corresponds to doubling and halving, respectively.

## 4. Discussion

SLC35A1-CDG stands out as one of the very rare glycosylation disorders, with only six reported cases to date, including the newly described patient. Commonly observed symptoms encompass seizures, microcephaly, ataxia, delayed development, and macrothrombocytopenia [33]. Notably, routine CDG diagnostics, which relies on glycan analysis of serum or plasma, does not consistently detect the presence of SLC35A1-CDG. This was also the case for our patient, who exhibited normal IEF patterns of transferrin, antitrypsin, and ApoCIII. Furthermore, only marginal abnormalities in lectin stainings and LC-MS analysis of whole serum N-glycans characterized by a 50% reduced level of the biantennary disialylated structure A2G2S2 and a general reduction of *α*-2,3 sialylated N-glycans were the major hallmarks. No specific biomarker for this type of disease was found. We assume that some patients with this defect will go undetected in CDG diagnostics, potentially contributing to the small number of identified patients so far.

However, it is important to highlight the reduced ADAMTS13 levels found in serum. ADAMTS13 is a protease responsible for cleaving von Willebrand factor (vWF) multimers in the blood. Inadequate functioning of ADAMTS13 can lead to Upshaw–Schulman syndrome, a disorder characterized by thrombotic thrombocytopenic purpura [34]. Thrombocytopenia with impaired megakaryocyte maturation and increased platelet clearance was also observed in a mouse model lacking Slc35a1 in megakaryocytes and platelets [35]. Ma et al. suggested that the detected diminished *α*-2,3 sialylation, which displays the primary form of platelet sialylation [36], is implicated in this process [35]. Notably, thrombocytopenia has also been described in various other types of CDG, including ALG1-, ALG8-, GALE-, GNE-, MPI-, PMM2-, and B4GALT1-CDG [37]. Therefore, further investigations into the glycosylation-dependent functioning of ADAMTS13 and the *α*-2,3 sialylation-dependent platelet homeostasis could potentially provide new insights into the coagulopathy abnormalities observed in CDG. More obvious abnormalities with regard to glycosylation emerged from examinations in cell culture. Western blot analyses of the cellular glycosylation markers ICAM1, TGN46, and GP130 showed significant deviations. Binding studies using lectins targeting sialic acid residues and nonsialylated structures as well as LC-MS measurements of N-glycans from fibroblasts confirmed a general hypoglycosylation in the patient cells. We attribute these abnormalities to the reduced import of CMP-Neu5Ac into the Golgi apparatus in the patient cells of approximately 65% compared to GDP-Fuc and UDP-Gal. Transfected HEK293 *SLC35A1* K.o. cells showed comparable reglycosylation of cellular glycosylation markers when expressing either the wild-type *SLC35A1*-cDNA or the construct carrying the patient variant. Moreover, both constructs restored WT-like levels of CMP-Neu5Ac in the patient fibroblasts as well as in the HEK293 *SLC35A1* K.o. cells. Together, these findings suggest that the functionality of the mutated transporter did not appear to be severely affected. Since the CHX treatment further displayed that the patient's protein was approximately 40% less stable than the control protein, we suspect that the reduced CMP-Neu5Ac transport into the Golgi was primarily caused by the decreased stability of SLC35A1 (p.Thr45Ala).

Furthermore, the observed accumulation of CMP-Neu5Ac in the patient's fibroblasts could result in feedback inhibition of GNE within sialic acid synthesis, potentially affecting UDP-GlcNAc levels. As UDP-GlcNAc serves not only as the precursor substrate for CMP-Neu5Ac synthesis but also as a direct substrate for OGT in protein O-GlcNAcylation, we investigated the potential impact of the SLC35A1 deficiency on UDP-GlcNAc-dependent O-GlcNAcylation. This revealed a significant reduction in this type of glycosylation, which we consider to be an additional secondary effect with implications for crucial cellular processes including transcription, translation, signal transduction, apoptosis, and metabolism [38]. Given that O-GlcNAcylation plays a role in regulating mitochondrial network homeostasis [39], the ATP level was determined and found to be significantly decreased in the patient cells. As the homeostatic regulation of systemic lipid uptake, storage, and release is also closely linked to O-GlcNAcylation [40], abnormal results emerged when measuring the lipid composition of the patient fibroblasts. The increased amount of the glycerol esters DAG and TAG suggests potential influences on cell signaling at the plasma membrane, glycerophospholipid biosynthesis in the membrane of the endoplasmic reticulum [41], reduced nutrient supply, increased inflammatory reactions, and general metabolic dysregulation [42]. Elevated Cer levels can lead, among other effects, to an increased formation of reactive oxygen species (ROS), a disruption of the electron transport chain, and the associated influence on the energy supply [43] which could represent an additional factor for the diminished ATP level found in the SLC35A1-deficient cells. Furthermore, an abnormal level of phosphoethanolamine PE-P, particularly present in neurons, muscle cells, platelets, neutrophils, and macrophages, might impact membrane fluidity, the regulation of exo- and endocytosis, and the binding of free radicals to protect cellular components through oxidative stress or cell division [44, 45]. Our data suggest that improper energy and lipid homeostasis negatively affect the proliferation and cell division of SLC35A1-CDG fibroblasts, an effect that was also detected in PMM2-CDG [46]. This disruption could ultimately contribute to the development of neurological and hematological issues encountered in SLC35A1-CDG and PMM2-CDG. It is noteworthy that proliferation and cell division were improved in SLC35A1-CDG when GlcNAc, a precursor metabolite of CMP-Neu5Ac, was added to the cell culture medium. Furthermore, GlcNAc supplementation reduced hypoglycosylation, as seen in Western and lectin blots. Although we did not follow the metabolic pathway of the provided GlcNAc, it seems likely that the sugar could be involved in glycosylation and energy metabolism, contributing to a beneficial cellular outcome. GlcNAc supplementation may therefore represent an alternative therapeutic concept to sialic acid and *N*-acetylmannosamine (ManNAc) administration [17] for SLC35A1-CDG, especially for patients with a comparatively high residual activity of the CMP-Neu5Ac transporter.

In conclusion, it is important to mention that SLC35A1-CDG can be missed in routine CDG diagnostics. Our work illustrates that—in some cases—the functional relevance of an uncharacterized genetic variant can only be detected through extensive laboratory analyses. Our investigations support the association of the variant c.133A>G (p.Thr45Ala), which mainly affects the protein stability of SLC35A1 negatively, with the biochemical phenotypes of SLC35A1-CDG. Our results show that hypoglycosylation was observed in the patient's fibroblasts, whereas the serum glycoproteins were mostly unaltered. This suggests a cell-specific phenotype of the mutated SLC35A1. However, it remains elusive to which extent the mutated transporter affects the glycosylation in other human tissues. Based on the described SLC35A1-CDG patients, we define seizures, microcephaly, ataxia, developmental delay, and macrothrombocytopenia as the more common clinical characteristics of this type of disease and add café-au-lait spot, ear tag, and umbilical hernia as possibly new associated phenotypes. However, as in the proband reported here, the relatively high residual SLC35A1 transporter activity may result in rather mild clinical features, which may even regress over the course of a comparatively benign disease progression. Finally, the supplementation of GlcNAc could represent a novel and easy-to-implement therapeutic approach for SLC35A1-CDG patients.

## Figures and Tables

**Table 1:** Overview of the yet-known six SLC35A1-CDG patients concerning clinical, biochemical, and genetic data.

	**Patient 1 (Willig et al. [** 31 **]; Martinez-Duncker et al. [** 15 **])**	**Patient 2 (Mohamed et al. [** 16 **])**	**Patient 3 (Ng et al. [** 17 **])**	**Patient 4 and Patient 5 (Kauskot et al. [** 14 **])**	**Patient 6 (this publication)**	
Gender	Male	Female	Female	Female, male	Female	
CDNA variant(s)	c.277_280delGTGCinsT/c.752-158_752-157insCACT	c.303G>C	c.467C>G/c.586G>A	c.439T>C	c.133A>G	
Protein variant(s)	p.Val93Cysfs∗17/p.Val208Phefs∗20	p.Gln101His	p.Thr156Arg/p.Glu196Lys	p.Ser147Pro	p.Thr45Ala	
Zygosity	Compound heterozygous	Homozygous	Compound heterozygous	Homozygous	Homozygous	
Protein domain	TMD 3 and 3rd cytoplasmic loop	TMD 3	TMD 5 and 6	TMD 5	1st lumenal loop	See Figure 1e
SLC35A1 activity	n.d.	App. 50%	App. 11%	n.d.	App. 66%	
Common symptoms						In total
Hypotonia	n.d.	Yes (lower extremities)	Yes (generalized muscular)	n.d.	No	2/6
Seizures	n.d.	Yes (tonic–clonic)	Yes (versive, with orofacial tics)	Yes (n.s.)	Yes (tonic–clonic, single event)	5/6
Microcephaly	n.d.	Yes	No	Yes	No	3/6
Ataxia	n.d.	Yes (mild)	Yes (mild)	Yes	No	4/6
Physical abnormalities	n.d.	Yes (facial features, clinodactyly 4th and 5th fingers on both hands, webbed neck, bil. hallux valgus, joint hyperlaxity)	n.d.	n.d.	Yes (café-au-lait, preauricular ear tag, small stature)	2/6
Development	n.d.	Delayed (psychomotor)	n.d.	Delayed (psychomotor)	Delayed (mild)	4/6
Macrothrombocytopenia	Yes	Yes	No	Yes	No^a^	4/6
Neutropenia	Yes	No	n.d.	n.d.	Yes	2/6
Hemorrhagic complications	Yes (lung, posterior chamber of the eye and cutaneous bleeding)	n.d.	n.d.	Yes (easy bruising, menorrhagia)	n.d.	2/6
Recurrent/opportunistic infections	Yes (leading to acute respiratory distress syndrome, pneumonia)	n.d.	No	n.d.	Yes	2/6
Behavioral problems	n.d.	Yes	Yes	n.d.	n.d.	2/6
Deceased	Yes (3 years)	Yes (after surgery, 22 years)	n.d.	n.d.	n.d.	2/6
Further symptoms	Lack of the sialyl-Lex antigen, CD15s on polymorphonuclear cells	Systolic cardiac murmur, proteinuria, aminoaciduria, low free thyroxine and estradiol, coagulopathy	Parietal arachnoidal cyst, IQ < 55, dysarthria, autistic features, thoracolumbar scoliosis	n.s.	Lactose intolerance, PFO (hemodynamically insignificant)	
Transferrin IEF, CE, MS (serum/plasma)	Normal	Abnormal	Abnormal	Abnormal	Normal	4/6
ApoCIII IEF, CE (serum/plasma)	Normal	Abnormal	n.d.	n.d.	Normal	1/6

Abbreviations: n.d. = not defined, n.s. = not specified.

^a^Thrombocytopenia was seen in early childhood.

## Data Availability

The data that support the findings of this study are available from the corresponding author upon reasonable request.

## Supporting Information

Supporting Information 1 Figure S1: Clustal Omega alignment of SLC35A1 around threonine45 of different species and analysis of reglycosylation of transfected HEK293 *SLC35A1* K.o. cells. (A) The protein comparison shows that both Thr45 (T45) and its adjacent amino acids (Y42, F43, S44, T46, and A47) are highly conserved across multiple species, suggesting an essential role of this amino acid for SLC35A1 function. (B) Western blot analysis of GP130 and TGN46 in HEK293 transfected with pCI-neo vector (WT Mock) and in CRISPR/Cas9 HEK293 *SLC35A1* knockout (K.o.) cells either transfected with pCI-neo vector (K.o. Mock), with the pCI-neo plasmid carrying the wild-type *SLC35A1* sequence (K.o. + WT) or the pCI-neo plasmid carrying the mutated *SLC35A1* gene (K.o. + A133G). K.o. cells expressing the wild-type *SLC35A1* showed reglycosylation (black arrow) of both marker proteins (Lane 3, respectively). Notably, reglycosylation was also achieved in the same manner by expression of *SLC35A1* carrying the c.A133G variant (Lane 4, respectively). (C) Determination of CMP-Neu5Ac level in fibroblasts (left) and HEK293 WT and *SLC35A1* K.o. cells transfected with pCI-neo vector (Mock), the pCI-neo plasmid carrying the wild-type *SLC35A1* gene (K.o. + WT) or the pCI-neo plasmid carrying the mutated *SLC35A1* (K.o. + A133G). Wild-type-like levels of CMP-Neu5Ac were obtained both when transfected with WT *SLC35A1* and with *SLC35A1* carrying the patient's variant (right).

Supporting Information 2 Figure S2: Analysis of serum glycoproteins. (A) Lectin staining of serum glycoproteins revealed a significant decrease in GlcNAc and *α*-2,3 sialylated glycans (WGA, MAL-I) in the patient's serum as well as a milder reduction of *α*-2,6 sialylated residues (SNA) compared to the serum control pool (control, *n* = 120). (B) Expression analysis revealed a significant reduction of the highly glycosylated ADAMTS13 in the patient's serum compared to the serum control pool (control, *n* = 120). (C, D) Whole serum glycoproteins were analyzed by LC-MS. The patient's sample showed elevated amounts of biantennary N-glycans of the complex type, such as F(6)A2(6)G(4)1 and A2G(4)2S(6)1 (see red arrows), along with a general 50% reduction in sugar structures, as observed in the biantennary sialylated complex-type structure A2G(4)2S(6,6)2.

Supporting Information 3 Figure S3: TGN46 and O-GlcNAcylation in fibroblasts. (A) Expression of the glycosylation marker protein TGN46 is significantly altered in the patient's cells (Pat.) compared to controls (Ctrl.). For quantification, the fully glycosylated protein forms (glyco., black arrow) were normalized to the hypoglycosylated forms (hypoglyco, grey arrow). (C) LC-MS analysis showed a similar distribution of N-glycans in the patient's serum compared to the serum control pool (control, *n* = 120). (B) The O-GlcNAcylation in the patient's fibroblasts is impaired as seen by a significantly reduced level of the O-GlcNAcylated form of histone H2B (H2B O-GlcNAc) and the general reduction of O-GlcNAcylated proteins in the patient's cell lysate which was also significantly altered (O-GlcNAc antibody and lectin succinylated [succ.] WGA). The reduced amount of O-GlcNAcylated proteins might be related to a decrease in the expression of O-GlcNAc transferase (OGT).

Supporting Information 4 Table S1: N-Glycans determined by LC-MS analysis in serum and fibroblasts. *N*-Glycans derived from whole serum glycoproteins and from skin fibroblasts of a healthy control and of the SLC35A1-CDG were analysed by LC-MS. Intensities of N-glycan peaks were calculated by normalizing to the sum of all assigned peaks. Structural assignment of N-glycans follows the Symbol Nomenclature for Glycans (SNFG) guidelines (Glycobiology 29:620-624, 2019; PMID 31184695).

Supporting Information 5 Table S2: Concentration of lipid classes in patient and control fibroblasts. Lipid concentrations were determined using ESI-MS/MS and normalized to cell numbers. For the graphical representation, the concentrations were normalized to the respective mean value of the control. Mean values, standard deviations, and the *p* value of Student's *t*-test after Sidak–Holm correction are listed. Abbreviations: Cer, ceramide; PE-P, phosphatidylethanolamine plasmalogen within the main lipid classes; TAG, triacylglycerol within the main lipid classes; CE, cholesteryl ester; Chol, cholesterol; DAG, diacylglycerol; PC, phosphatidylcholine; PE, phosphatidylethanolamine; PI, phosphatidylinositol; PS, phosphatidylserine; SM, sphingomyelin; Hex2Cer, dihexosylceramide; HexCer, hexosylceramide; LPC, lysophosphatidylcholine; PA, phosphatidic acid; PG, phosphatidylglycerol.
