# Supplementary material for: A Novel Missense Variant in Ultrarare SLC35A1-CDG Alters Cellular Glycosylation, Lipid, and Energy Metabolism Without Affecting CDG Serum Markers
Source: Hum Mutat. 2025 Jun 26;2025:6290620. doi: 10.1155/humu/6290620 (PMC12226171; doi:10.1155/humu/6290620)
Supplement: Supporting Information 1 — Figure S1: Clustal Omega alignment of SLC35A1 around threonine45 of different species and analysis of reglycosylation of transfected HEK293 SLC35A1 K.o. cells. (A) The protein comparison shows that both Thr45 (T45) and its adjacent amino acids (Y42, F43, S44, T46, and A47) are highly conserved across multiple species, suggesting an essential role of this amino acid for SLC35A1 function. (B) Western blot analysis of GP130 and TGN46 in HEK293 transfected with pCI-neo vector (WT Mock) and in CRISPR/Cas9 HEK293 SLC35A1 knockout (K.o.) cells either transfected with pCI-neo vector (K.o. Mock), with the pCI-neo plasmid carrying the wild-type SLC35A1 sequence (K.o. + WT) or the pCI-neo plasmid carrying the mutated SLC35A1 gene (K.o. + A133G). K.o. cells expressing the wild-type SLC35A1 showed reglycosylation (black arrow) of both marker proteins (Lane 3, respectively). Notably, reglycosylation was also achieved in the same manner by expression of SLC35A1 carrying the c.A133G variant (Lane 4, respectively). (C) Determination of CMP-Neu5Ac level in fibroblasts (left) and HEK293 WT and SLC35A1 K.o. cells transfected with pCI-neo vector (Mock), the pCI-neo plasmid carrying the wild-type SLC35A1 gene (K.o. + WT) or the pCI-neo plasmid carrying the mutated SLC35A1 (K.o. + A133G). Wild-type-like levels of CMP-Neu5Ac were obtained both when transfected with WT SLC35A1 and with SLC35A1 carrying the patient's variant (right). [file 6290620.f1.pptx]

## Slide 1
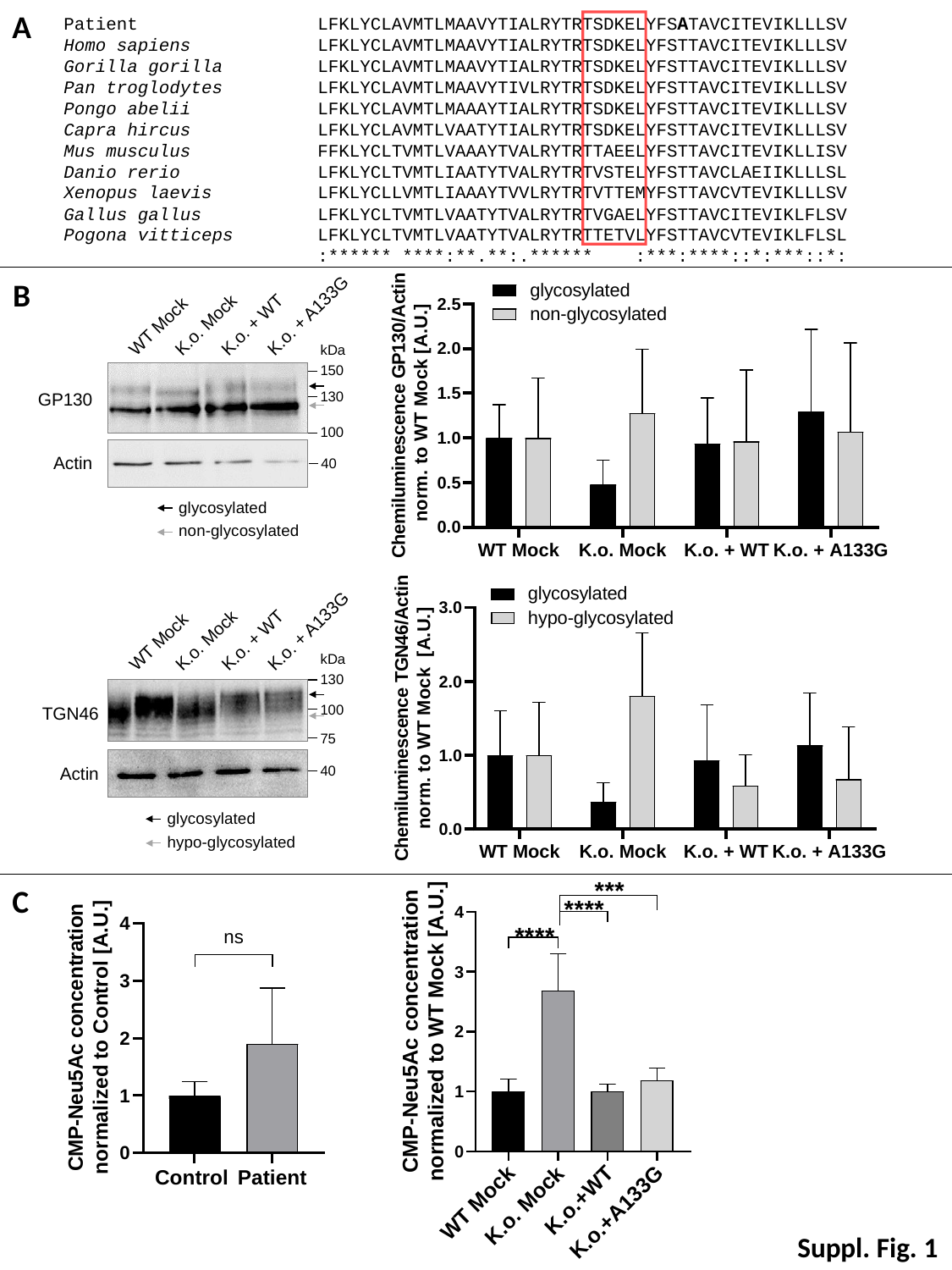

A
Patient 		LFKLYCLAVMTLMAAVYTIALRYTRTSDKELYFSATAVCITEVIKLLLSV 	60
Homo sapiens 	LFKLYCLAVMTLMAAVYTIALRYTRTSDKELYFSTTAVCITEVIKLLLSV 	60
Gorilla gorilla 	LFKLYCLAVMTLMAAVYTIALRYTRTSDKELYFSTTAVCITEVIKLLLSV 	60
Pan troglodytes 	LFKLYCLAVMTLMAAVYTIVLRYTRTSDKELYFSTTAVCITEVIKLLLSV 	60
Pongo abelii 	LFKLYCLAVMTLMAAAYTIALRYTRTSDKELYFSTTAVCITEVIKLLLSV 	60
Capra hircus 	LFKLYCLAVMTLVAATYTIALRYTRTSDKELYFSTTAVCITEVIKLLLSV 	60
Mus musculus 	FFKLYCLTVMTLVAAAYTVALRYTRTTAEELYFSTTAVCITEVIKLLISV 	60
Danio rerio 	LFKLYCLTVMTLIAATYTVALRYTRTVSTELYFSTTAVCLAEIIKLLLSL 	58
Xenopus laevis 	LFKLYCLLVMTLIAAAYTVVLRYTRTVTTEMYFSTTAVCVTEVIKLLLSV 	59
Gallus gallus 	LFKLYCLTVMTLVAATYTVALRYTRTVGAELYFSTTAVCITEVIKLFLSV 	60
Pogona vitticeps	LFKLYCLTVMTLVAATYTVALRYTRTTETVLYFSTTAVCVTEVIKLFLSL 	60
 		:****** ****:**.**:.****** :***:****::*:***::*:
B
WT Mock
K.o. Mock
K.o. + WT
K.o. + A133G
kDa
150
130
GP130
100
Actin
40
glycosylated
non-glycosylated
WT Mock
K.o. Mock
K.o. + WT
K.o. + A133G
kDa
130
100
TGN46
75
40
Actin
glycosylated
hypo-glycosylated
C
Suppl. Fig. 1
