# Supplementary material for: A Novel Missense Variant in Ultrarare SLC35A1-CDG Alters Cellular Glycosylation, Lipid, and Energy Metabolism Without Affecting CDG Serum Markers
Source: Hum Mutat. 2025 Jun 26;2025:6290620. doi: 10.1155/humu/6290620 (PMC12226171; doi:10.1155/humu/6290620)
Supplement: Supporting Information 2 — Figure S2: Analysis of serum glycoproteins. (A) Lectin staining of serum glycoproteins revealed a significant decrease in GlcNAc and α-2,3 sialylated glycans (WGA, MAL-I) in the patient's serum as well as a milder reduction of α-2,6 sialylated residues (SNA) compared to the serum control pool (control, n = 120). (B) Expression analysis revealed a significant reduction of the highly glycosylated ADAMTS13 in the patient's serum compared to the serum control pool (control, n = 120). (C, D) Whole serum glycoproteins were analyzed by LC-MS. The patient's sample showed elevated amounts of biantennary N-glycans of the complex type, such as F(6)A2(6)G(4)1 and A2G(4)2S(6)1 (see red arrows), along with a general 50% reduction in sugar structures, as observed in the biantennary sialylated complex-type structure A2G(4)2S(6,6)2. [file 6290620.f2.pptx]

## Slide 1
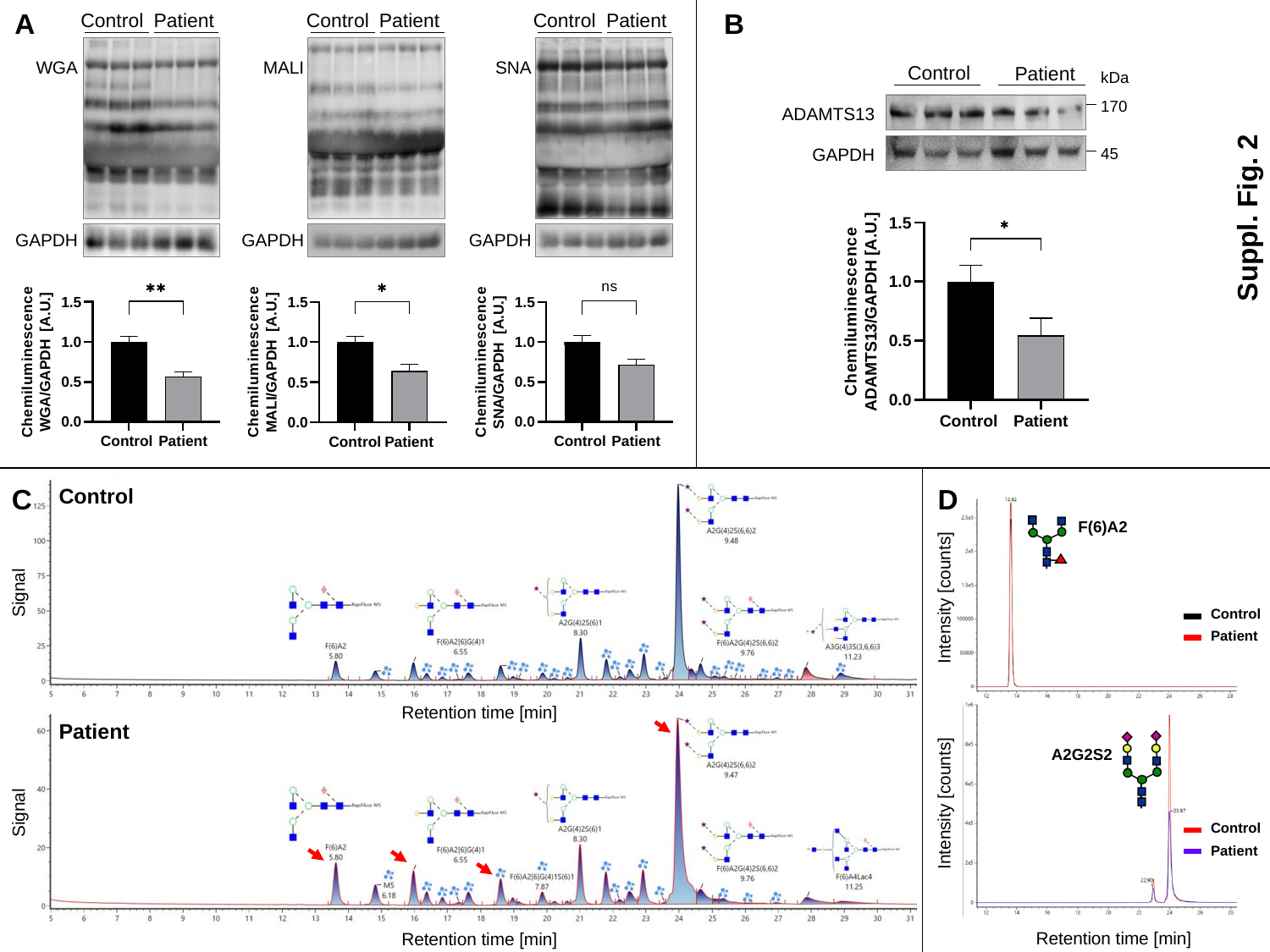

A
B
Control
Patient
Control
Patient
Control
Patient
WGA
MALI
SNA
Control
Patient
kDa
170
ADAMTS13
45
GAPDH
Suppl. Fig. 2
GAPDH
GAPDH
GAPDH
Control
Signal
Retention time [min]
Patient
Signal
Retention time [min]
C
D
F(6)A2
Intensity [counts]
Control
Patient
A2G2S2
Intensity [counts]
Control
Patient
Retention time [min]
