# Supplementary material for: A Novel Missense Variant in Ultrarare SLC35A1-CDG Alters Cellular Glycosylation, Lipid, and Energy Metabolism Without Affecting CDG Serum Markers
Source: Hum Mutat. 2025 Jun 26;2025:6290620. doi: 10.1155/humu/6290620 (PMC12226171; doi:10.1155/humu/6290620)
Supplement: Supporting Information 3 — Figure S3: TGN46 and O-GlcNAcylation in fibroblasts. (A) Expression of the glycosylation marker protein TGN46 is significantly altered in the patient's cells (Pat.) compared to controls (Ctrl.). For quantification, the fully glycosylated protein forms (glyco., black arrow) were normalized to the hypoglycosylated forms (hypoglyco, grey arrow). (C) LC-MS analysis showed a similar distribution of N-glycans in the patient's serum compared to the serum control pool (control, n = 120). (B) The O-GlcNAcylation in the patient's fibroblasts is impaired as seen by a significantly reduced level of the O-GlcNAcylated form of histone H2B (H2B O-GlcNAc) and the general reduction of O-GlcNAcylated proteins in the patient's cell lysate which was also significantly altered (O-GlcNAc antibody and lectin succinylated [succ.] WGA). The reduced amount of O-GlcNAcylated proteins might be related to a decrease in the expression of O-GlcNAc transferase (OGT). [file 6290620.f3.pptx]

## Slide 1
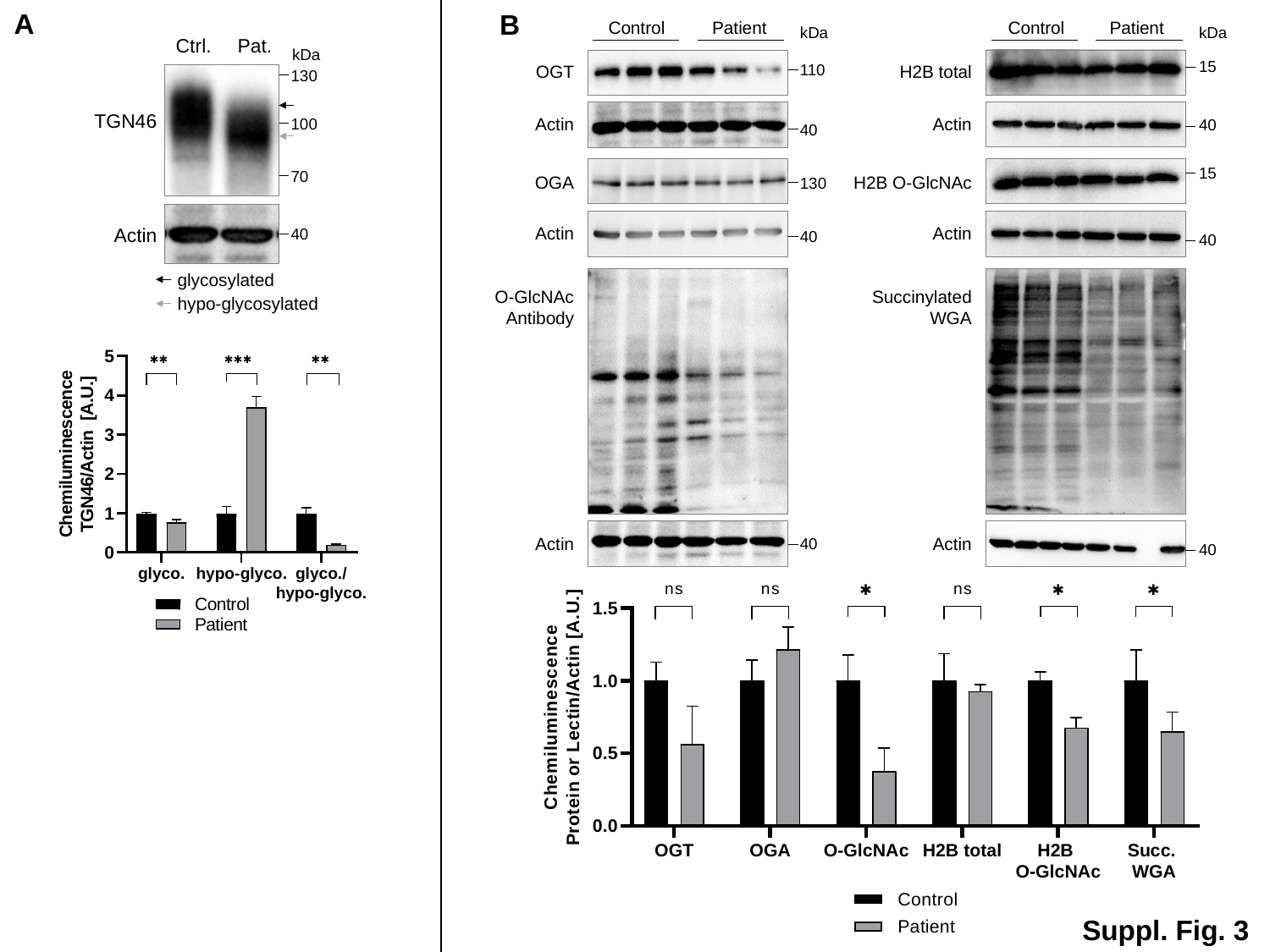

A
B
Control
Patient
Control
Patient
kDa
kDa
Ctrl.
Pat.
kDa
130
TGN46
100
70
Actin
40
glycosylated
hypo-glycosylated
15
110
OGT
H2B total
Actin
Actin
40
40
15
OGA
H2B O-GlcNAc
130
Actin
Actin
40
40
O-GlcNAc
Antibody
Succinylated WGA
Actin
Actin
40
40
Suppl. Fig. 3
