# Supplementary material for: A Novel Missense Variant in Ultrarare SLC35A1-CDG Alters Cellular Glycosylation, Lipid, and Energy Metabolism Without Affecting CDG Serum Markers
Source: Hum Mutat. 2025 Jun 26;2025:6290620. doi: 10.1155/humu/6290620 (PMC12226171; doi:10.1155/humu/6290620)
Supplement: Supporting Information 5 — Table S2: Concentration of lipid classes in patient and control fibroblasts. Lipid concentrations were determined using ESI-MS/MS and normalized to cell numbers. For the graphical representation, the concentrations were normalized to the respective mean value of the control. Mean values, standard deviations, and the p value of Student's t-test after Sidak–Holm correction are listed. Abbreviations: Cer, ceramide; PE-P, phosphatidylethanolamine plasmalogen within the main lipid classes; TAG, triacylglycerol within the main lipid classes; CE, cholesteryl ester; Chol, cholesterol; DAG, diacylglycerol; PC, phosphatidylcholine; PE, phosphatidylethanolamine; PI, phosphatidylinositol; PS, phosphatidylserine; SM, sphingomyelin; Hex2Cer, dihexosylceramide; HexCer, hexosylceramide; LPC, lysophosphatidylcholine; PA, phosphatidic acid; PG, phosphatidylglycerol. [file 6290620.f5.docx]

| **Average values and standard deviation [µM]** | | | | | **Normalization to the average values of the control (increased x-fold)** | | | |  |
| --- | --- | --- | --- | --- | --- | --- | --- | --- | --- |
| **Lipid** | **Mean (control)** | **standard deviation (control)** | **Mean (patient)** | **standard deviation (patient)** | **control** | **standard deviation (control)** | **patient** | **standard deviation (patient)** | **p-value** |
| **CE** | 7,80 | 0,78 | 7,06 | 2,16 | 1,00 | 0,10 | 0,91 | 0,28 | 0,991 |
| **Cer** | 1,05 | 0,09 | 8,10 | 1,48 | 1,00 | 0,08 | 7,74 | 1,41 | 0,002 ** |
| **Chol** | 104,87 | 9,26 | 164,48 | 44,23 | 1,00 | 0,09 | 1,57 | 0,42 | 0,401 |
| **DAG** | 1,98 | 0,24 | 6,42 | 2,67 | 1,00 | 0,12 | 3,23 | 1,35 | 0,245 |
| **Hex2Cer** | 0,49 | 0,05 | 0,65 | 0,17 | 1,00 | 0,11 | 1,32 | 0,35 | 0,722 |
| **HexCer** | 1,49 | 0,16 | 3,12 | 0,80 | 1,00 | 0,11 | 2,09 | 0,53 | 0,127 |
| **LPC** | 2,91 | 0,31 | 2,85 | 0,83 | 1,00 | 0,11 | 0,98 | 0,29 | 0,991 |
| **PA** | 1,40 | 0,21 | 1,54 | 0,48 | 1,00 | 0,15 | 1,10 | 0,34 | 0,991 |
| **PA O** | 0,07 | 0,01 | 0,09 | 0,03 | 1,00 | 0,14 | 1,24 | 0,41 | 0,918 |
| **PC O** | 15,07 | 1,07 | 21,91 | 5,27 | 1,00 | 0,07 | 1,45 | 0,35 | 0,417 |
| **PC** | 118,25 | 10,08 | 155,39 | 39,55 | 1,00 | 0,09 | 1,31 | 0,33 | 0,717 |
| **PE** | 66,58 | 5,42 | 73,04 | 19,94 | 1,00 | 0,08 | 1,10 | 0,30 | 0,991 |
| **PE O** | 8,53 | 0,89 | 15,37 | 4,50 | 1,00 | 0,10 | 1,80 | 0,53 | 0,295 |
| **PE P** | 20,34 | 1,53 | 45,92 | 4,63 | 1,00 | 0,08 | 2,26 | 0,23 | 0,001 *** |
| **PG** | 0,76 | 0,14 | 1,36 | 0,48 | 1,00 | 0,19 | 1,78 | 0,63 | 0,463 |
| **PG O** | 0,05 | 0,00 | 0,05 | 0,02 | 1,00 | 0,07 | 1,05 | 0,39 | 0,991 |
| **PI** | 14,28 | 1,38 | 17,10 | 4,41 | 1,00 | 0,10 | 1,20 | 0,31 | 0,918 |
| **PI O** | 0,44 | 0,06 | 0,47 | 0,13 | 1,00 | 0,14 | 1,08 | 0,30 | 0,991 |
| **PS** | 20,85 | 1,45 | 35,30 | 9,29 | 1,00 | 0,07 | 1,69 | 0,45 | 0,282 |
| **PS O** | 1,45 | 0,11 | 2,89 | 0,78 | 1,00 | 0,08 | 1,99 | 0,53 | 0,172 |
| **SM** | 18,33 | 0,85 | 30,46 | 7,45 | 1,00 | 0,05 | 1,66 | 0,41 | 0,250 |
| **TAG** | 3,82 | 1,87 | 16,04 | 4,46 | 1,00 | 0,49 | 4,20 | 1,17 | 0,045 * |
|  |  |  |  |  |  |  |  |  |  |

**Supplemental table 1: Concentration of lipid classes in patient and control fibroblasts.** Lipid concentrations were determined using ESI-MS/MS and normalized to cell numbers. For the graphical representation, the concentrations were normalized to the respective mean value of the control. Mean values, standard deviations and the p-value of the Student's t-test after Sidak-Holm correction are listed. Abbreviations for lipids can be found under Figure legends.
